# Antioxidant and Antiproliferative Potential of Bioactive Molecules Ursolic Acid and Thujone Isolated from *Memecylon edule* and *Elaeagnus indica* and Their Inhibitory Effect on Topoisomerase II by Molecular Docking Approach

**DOI:** 10.1155/2020/8716927

**Published:** 2020-02-14

**Authors:** Ramalingam Srinivasan, Arumugam Aruna, Jong Suk Lee, Myunghee Kim, Muthugounder Subramaniam Shivakumar, Devarajan Natarajan

**Affiliations:** ^1^Department of Food Science and Technology, Yeungnam University, Gyeongsan-si, Gyeongsangbuk-do 38541, Republic of Korea; ^2^Department of Biotechnology, K. S. Rangasamy College of Arts and Science, K. S. R. Kalvi Nagar, Tiruchengode 637215, Namakkal, Tamil Nadu, India; ^3^Department of Biotechnology, Periyar University, Salem 636 011, Tamil Nadu, India; ^4^Department of Food & Nutrition & Cook, Taegu Science University, Daegu 41453, Republic of Korea

## Abstract

The present study aimed to evaluate the antioxidant and antiproliferative potential of ursolic acid and thujone isolated from leaves of *Elaeagnus indica* and *Memecylon edule* and their inhibitory effect on topoisomerase II using molecular docking study. The isolated ursolic acid and thujone were examined for different types of free radicals scavenging activity, the antiproliferative potential on U-937 and HT-60 cell lines by adopting standard methods. Further, these compounds were docked with the active site of the ATPase region of topoisomerase II. The findings of the research revealed that ursolic acid harbor strong antioxidant and antiproliferative capacity with low IC_50_ values than the thujone in all tested methods. Moreover, ursolic acid shows significant inhibition effect on topoisomerase II with a considerable docking score (−8.0312) and GLIDE energy (−51.86 kca/mol). The present outcome concludes that ursolic acid possesses significant antioxidant and antiproliferative potential, which can be used in the development of novel antioxidant and antiproliferative agents in the future.

## 1. Introduction

Free radicals are involved in numerous cellular functions including defense mechanisms and cell signaling and are essential for the aerobic metabolism. However, the overproduction of free radicals in cells leads to oxidative stress, consequently causing damage to vital macromolecules, like DNA, lipids, and proteins [1]. Several degenerative related diseases such as cancer, inflammation, atherosclerosis, cataracts, asthma, diabetes mellitus anemia, brain dysfunction, arthritis, liver diseases, and renal problems are predominantly linked to the oxidative stress [2].

Cancer is the malignant illness and the second most leading cause of mortality worldwide. Globally, various types of cancer caused 9.6 million deaths in 2018, approximately 1 in 6 deaths. The people of low- and middle-income countries are highly affected by cancer, due to the change in behavior and diet, and the estimates were around 70% of deaths [3]. In western countries, hormone-dependent cancers such as breast, prostate, and uterine cervix are a common cause of several deaths [4]. Nowadays, different treatments such as chemotherapy, surgery, radiotherapy, and antihormone therapy are used to treat cancer. However, these therapies are expensive and cause an adverse effect on host health. Several plant-derived compounds like vinblastine, vincristine, taxol, and camptothecin were used in the treatment of cancer [5]. Thus, various research groups around the world have focused on the investigation of plant extract to find a plant-based novel, broad-spectrum, cost-effective, better, and safer anticancer and antioxidant agent from plant materials [6].

*Elaeagnus indica* (Elaeagnaceae) and *Memecylon edule* (Melastomataceae) are known to possess various ethnobotanical properties and used to treat various ailments in traditional medicine [2, 7, 8]. Both plants were reported to have various biological activities, including larvicidal, antibacterial, anti-inflammatory, analgesic, antioxidant, and anticancer activities, and few compounds were identified [1, 2, 7–17]. However, there is no data available on the antioxidant and antiproliferative properties of the isolated compound thujone (from *Elaeagnus indica*). Even though anticancer and antiproliferative activity of ursolic acid (isolated from *Memecylon edule*) were reported [18–21], there is no data available on growth inhibitory effect of ursolic acid on the human leukemic monocyte lymphoma (U-937) cells, and antioxidant potential of ursolic acid was the least explored. Moreover, there are no previous studies available on the *in silico* inhibition interactions of thujone and ursolic acid with topoisomerase II in detail. Thus, the present investigation aimed to examine the antioxidant and antiproliferative potential of ursolic acid and thujone isolated from leaves of *Elaeagnus indica* and *Memecylon edule* and evaluate their inhibitory effect on the topoisomerase II using molecular docking tools.

## 2. Materials and Methods

### 2.1. Plant Materials

Fresh and healthy aerial parts of *Elaeagnus indica* and *Memecylon edule* were collected from different regions of Shervarayan Hills (latitude 11°47′−12°33′ N, longitude 77°02′−78°40′ E, 1300–400 m MSL), Salem District, and Kolli Hills (latitude 10°12′−11°07′ N, longitude 76°−77°17′ E, 900–1100 m MSL), Namakkal District, Tamil Nadu, India, respectively. The nomenclature of collected plant material was authenticated by the Botanical Survey of India (BSI) (*E*. *indica* reference letter No. BSI/SRC/5/23/2014–15/Tech/1942 and *M*. *edule* reference letter No. BSI/SRC/5/23/2014–15/Tech./248) Coimbatore, Tamil Nadu, India. Herbarium specimens of collected plants were deposited (*E*. *indica* specimen No. PU/DBT/NDRL//2010/03 and *M*. *edule* specimen No. PU/DBT/NDRL//2010/05) in Natural Drug Research Laboratory (NDRL), Department of Biotechnology, Periyar University, Salem, Tamil Nadu, India. The plant materials were washed with the running water prior to sterile distilled water and air-dried at room temperature for 14–21 days. The dried plant materials were pulverized, using an electric grinder, and then sieved through 100-mesh sifter and stored in an airtight container for further use.

### 2.2. Extraction of Plant Materials

Pulverized plant materials (2 kg) were successively extracted with various organic solvents such as hexane, chloroform, ethyl acetate, acetone, and methanol (1 : 5 solvent ratio) in an increasing polarity manner using a Soxhlet apparatus until the efflux solvents become colorless. The extracts were filtered through Whatman No. 1 filter paper and condensed using a rotary evaporator in vacuum at 40°C which yields greenish crude extracts. These extracts were stored in an airtight container at 4°C until use.

### 2.3. Isolation of Bioactive Molecules

Based on the preliminary results of phytochemical profile and biological activity [7, 8, 14–17], two extracts (namely acetone extract of *E*. *indica* and the ethyl acetate extract of *M*. *edule*) were selected for the isolation of active principles. The activity guided isolation of extract yields two active compounds [15, 17]. These compounds were identified using various spectral studies, like UV, FT-IR, LCMS, ^1^H, 13C, DEPT-135, HMBC, and HSQC Nuclear Magnetic Resonance [15, 17].

### 2.4. In Vitro Antioxidant Studies

Antioxidant potential of isolated compounds were examined on different types of free radicals, that is, DPPH, nitric oxide, hydroxyl, and superoxide radical and ferric reducing antioxidant power assay (FRAP) as per the previous standard methods [22–25]. Various concentrations (20–100 *µ*g/mL) of the isolated compounds were used in the radical scavenging potential analysis. Similar concentrations of a natural (ascorbic acid) and synthetic compound butylated hydroxyanisole (BHA) were used as reference molecules in all antioxidant investigations.

### 2.5. Antiproliferative Activity

The human leukemic monocyte lymphoma (U-937) and human acute promyelocytic leukemia (HT-60) cell lines were acquired from the National Institute of Cell Sciences, Pune, India, and maintained in Minimal Essential Medium (MEM) supplemented with 10% (v/v) heat-inactivated Fetal Bovine Serum (FBS), 3% L-glutamine, 100 IU/mL penicillin *G*, and 100 *µ*g/mL streptomycin in a 5% CO_2_ incubator at 37°C. The antiproliferative potential of isolated compounds was detected by methyl thiazolyl diphenyl-tetrazolium bromide (MTT) by adopting the method of Srinivasan et al. [16] on U-937 and HT-60 cell lines.

### 2.6. Molecular Docking Studies

Molecular interactions of ursolic acid and thujone with topoisomerase II (PDB id: 1QZR) were studied using *GLIDE* (*Grid-Based Ligand Docking with Energetics*) [26] software v5.5 developed by Schrödinger executed on Red Hat Enterprise Linux 5. *Maestro* v9.0 used for the preparation of ligands and proteins and docking study was carried out in Graphical User Interface (GUI, Maestro, 2009) workspace. *LigPrep* (Schrödinger suite, 2009) module of v2.3 of Schrödinger Suite 2009 was used to prepare the ligands. The energy minimization of *LigPrep* follows the optimized potential liquid simulations for all-atom force fields. Induced fit docking (IFD) of the prepared ligands with target proteins was done in *Induced Fit Docking* protocol of *GLIDE* v5.5 (Schrödinger Suite 2009). The images of docked complexes and hydrogen bond interactions were analyzed in the *PyMol* Molecular Graphics System. *Ligplot* diagram of docked complexes was obtained from PDBsum server (http://www.ebi.ac.uk/pdbsum) for better clarity.

#### 2.6.1. Preparation of Topoisomerase II

The crystal structure of the 1QZR complex (ATPase region of topoisomerase II from *Saccharomyces cerevisiae*) was retrieved from PDB which contains two identical 418 amino acid length polypeptide chains (A and B) with one (s)-4, 4′-(-1-methyl-1,2-ethanediyl) bis-2,6-piperazinedione (CDX), phosphoaminophosphonic acid-adenylate ester (ANP), and magnesium ion in each chain. Two methods were used in the preparation of protein for docking study. In the first method, all the water molecules, one CDX, one magnesium ion, and one ANP were removed and in the second method the the water molecules present in 1QZR were removed.

### 2.7. Statistical Analyses

All the analyses used in the present research were carried out in triplicate. Data were represented as the mean ± standard deviation of three quotients. The inhibitory concentrations 50 (IC_50_) were calculated by the curve fitted method using OriginPro 8 software. The significant difference was obtained from the results of Tukey's test (*p* < 0.05) of ANOVA (SPSS 25.0).

## 3. Results and Discussion

3*β*-hydroxyurs-12-en-28-oic acid (ursolic acid, a pentacyclic triterpenoid) and 1-isopropyl-4-methylbicyclo [3.1.0] hexan-3-one (thujone, a monoterpene ketone) were isolated from *Memecylon edule* and *Elaeagnus indica,* respectively, through activity guided isolation methods. Those compounds' isolation and structural elucidation were reported [15, 17]. Previously, these isolated compounds were reported from different parts of several plants [15–18].

### 3.1. In Vitro Antioxidant Studies

Both the isolated compounds ursolic acid and thujone expressed good to moderate radical scavenging activity in all tested methods in a concentration-dependent manner (Tables 1–5). Ursolic acid exhibited significant free radical scavenging activity on all tested radicals with the lowest IC_50_/EC_50_ values followed by thujone with sustainable IC_50_ values. Ursolic acid expressed good Fe^3+^ reduction potential in FRAP assay with the least EC_50_ value 18.42 ± 0.03 *µ*g/mL (Table 2) followed by hydroxyl radicals (IC_50_ value 29.69 ± 0.44 *µ*g/mL) (Table 3). NO radical scavenging potential of ursolic acid and thujone is almost similar to high IC_50_ value of 70.40 ± 0.88 µg/mL and 77.68 ± 0.58 *µ*g/mL, respectively, which are 2-fold higher than the positive control ascorbic acid (Table 4). Both the ursolic acid (76.92 %) and thujone (72.79 %) show nearly similar percentage of DPPH radical scavenging potential. However, considerable difference was found in the IC_50_ values (Table 1). Ursolic acid expressed a significantly high superoxide radical scavenging ability with low IC_50_ value of 43.35 ± 0.95 *µ*g/mL, which is lower than both natural and synthetic antioxidant controls, namely, ascorbic acid and BHA, respectively (Table 5). Thujone show the lowest quenching ability on the superoxide radicals with high IC_50_ value of 131.78 ± 1.27 *µ*g/mL (Table 6). The results of the antioxidant potential of thujone revealed that IC_50_ values of all radical scavenging activity were two- to threefold higher than the isolated ursolic acid when compared with reference compounds in most of the tested methods. Maximum antiradical activity was found in FRAP method with of EC_50_ value of 44.58 ± 0.89 *µ*g/mL. The results of the rest of assays expressed considerable radical scavenging potential of thujone.

The present investigation used 5 different assays to assess the antioxidant potential of ursolic acid and thujone. Both ursolic acid and thujone expressed different degree of radical scavenging potential on various tested radicals. The difference in the antioxidant capacity of the tested compounds obtained by the results of FRAP, DPPH radical, hydroxyl radical, nitric oxide radical, and superoxide radical scavenging methods is probably a result of the variation in sensitivity of isolated compounds to the various species of radicals [27]. Moreover, the variation in the reaction media such as lipophilic, hydrophilic, and amphiphilic and nature of radicals and antioxidant molecules have impact on the antiradical potential. For example, DPPH radicals are used to evaluate the antiradical potential of both hydrophilic and hydrophobic antioxidants, whereas nitric oxide assay is used for hydrophilic antioxidants [27]. The difference in the antioxidant potential of ursolic acid and thujone is due to the variation in their structure and functional groups such as free hydroxyl moiety and polarity.

There are no studies/reports on the antioxidant activity of thujone. However, several reports are present on the antioxidant activity of essential oils from various plants, that is, *Salvia officinalis* [28], *Artemisia herba-alba* [29], *Artemisia japonica*, *Artemisia nilagirica* [30], and *Artemisia absinthium* [31], which contains high amount of thujone. Based on the results of the aforementioned studies, thujone exhibited low to moderate antioxidant activities of all tested radicals. Mighri et al. [32] reported that thujone-rich oil from *Artemisia herba-alba* showed the lower inhibition percentage of antioxidant activity than the positive control (BHA) which strengthens the outcome of the present study. Earlier study on the antioxidant potential of ursolic acid shows similar IC_50_ value for the DPPH radicals scavenging activity [33] which supports the findings of the present investigation.

### 3.2. Antiproliferative Activity

**Figure 1:**
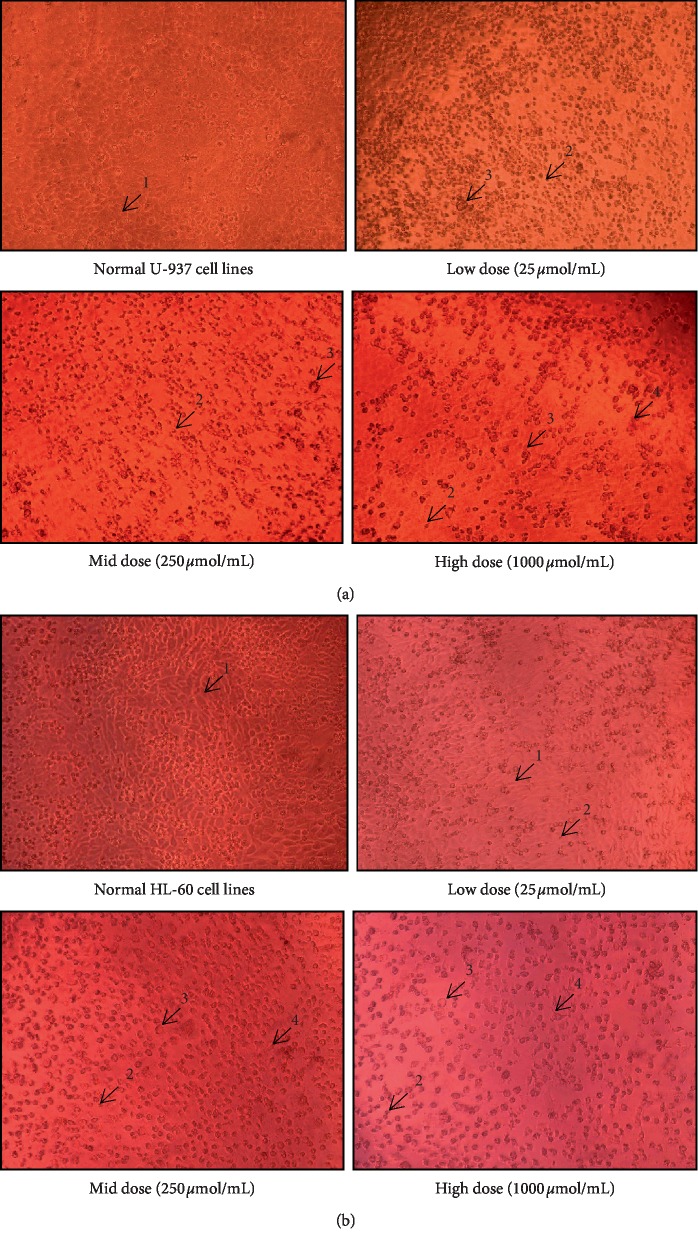
Antiproliferative potential (a and b) of isolated thujone (1-normal cells, 2-cell shrinkage, 3-membrane blebbing, 4-echinoid spikes, and 5-anoikis). (a) Antiproliferative effect of isolated thujone on U-937 cell line. (b) Antiproliferative effect of isolated ursolic acid on HL-60 cell line.

**Figure 2:**
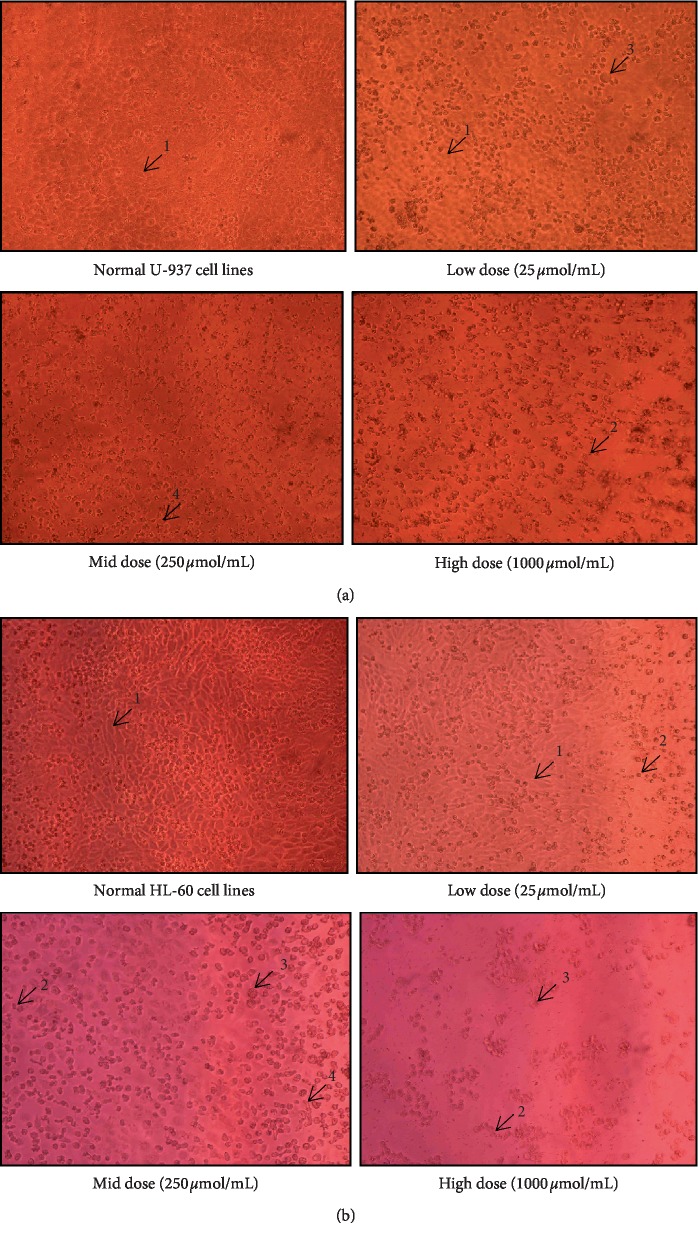
Antiproliferative potential (a and b) of isolated ursolic acid (1-normal cells, 2-cell shrinkage, 3-membrane blebbing, and 4-echinoid spikes). (a) Antiproliferative effect of isolated ursolic acid on U-937 cell line. (b) Antiproliferative effect of isolated ursolic acid on HL-60 cell line.

The results of the antiproliferative activity of ursolic acid and thujone expressed notable growth inhibitory effect on both U937 and HL-60 cells in a dose-dependent manner (Table 7). The present study shows ursolic acid harbor higher antiproliferative potential on both U-937 and HL-60 cells than the thujone. Furthermore, the findings of the present investigation revealed that HL-60 cells were more sensitive to ursolic acid than U-937 cells (Figure 1). Ursolic acid possessed a profound inhibitory effect on the proliferation of HL-60 cells (∼77% growth inhibition) with the lowest IC_50_ value (26.83 ± 3.07 *µ*mol/mL) followed by U-937 cells (∼80% growth inhibition) with considerable IC_50_ value (36.59 ± 0.80 *µ*mol/mL). Thujone shows moderate antiproliferative potential on U-937 cells (∼63% growth inhibition) with a high IC_50_ value of 297.42 ± 1.64 *µ*mol/mL (Figure 2). The least cytotoxic activity on HL-60 cell line (∼56% growth inhibition) with a higher IC_50_ value of 486.15 ± 2.74 *µ*mol/mL was noticed in thujone.

The total number of U937 and HL-60 cells was decreased with increased dose of both ursolic acid and thujone. Distinctive morphological changes in the U937 and HL-60 cells occurred with increasing concentration of ursolic acid and thujone treatment (Figures 1 and 2). Typical apoptotic features such as cell shrinkage and membrane blebbing were found in the U937 and HL-60 cells upon the low- to mid- and/or high-dose treatment of ursolic acid and thujone [27]. The treatment of ursolic acid and thujone induced the loss of cellular adhesion, echinoid spikes, and blistering. Moreover, the cells are detached from their basal membrane (anoikis) and lost their contact with adjacent cells upon treatment of ursolic acid and thujone at high dose. The increasing concentration of ursolic acid and thujone induced cell death through the necrosis process which, detected with characteristic morphological changes of necrosis in U937 and HL-60 cells such as membrane bubbling, detached from neighbor cells and evaginations [28]. Similarly, morphological changes were reported in the ursolic acid-treated various cancer cells that support the present findings [18–20, 33–36].

Ursolic acid is reported to have antiproliferative activity on different types of cancers such as lung, colon, breast, renal, prostate, melanoma, and leukemia [18–20, 33–36]. Many reports support the antiproliferative potential of ursolic acid because it might induce the apoptosis process in different mode on various types of cell lines [37, 38]. Previously, the antiproliferative activity of ursolic acid on HL-60 cell line was well-reported. It mainly induced apoptosis in HL-60 leukemia cells accompanied by mediating the release of intracellular calcium ions [39], inhibiting DNA synthesis [40] and blocking the cell cycle process [39, 41], and inducing Atg5-dependent autophagy [42] and mitochondria dependent apoptosis [19] which supports the findings of the present study. Moreover, many reviews described the mode of action of ursolic acid in the control of cancer cells [18–21].

Biswas et al. [43] evaluated the antiproliferative and apoptosis-inducing properties of thujone-rich fraction separated from *Thuja occidentalis* on melanoma (A375) cell line harbor higher IC_50_ (226.18 *μ*g/mL) value. Moreover, the thujone-rich fraction displayed least (∼14%) cytotoxicity on normal peripheral blood mononuclear cell (PBMC) [44] (Biswas et al., 2010). Similarly, Zolotovich et al. [44] reported that thujone exhibits no cytotoxic effect on HeLa cells at high concentrations (100 *µ*g/mL), which supports the outcome of present study. Likewise, Privitera et al. [5] documented that thujone-rich essential oil from *Salvia officinalis* has no cytotoxic effect on the LNCaP cells in all tested concentrations that strengthen the present findings.

### 3.3. Molecular Docking Studies

DNA topoisomerases are ubiquitous enzymes that unwind DNA molecule which is necessary for various DNA dependent biological processes [45]. Generally, two types of topoisomerases are predominantly found in cells, namely, type I and type II. Type I topoisomerases break single strand of duplex DNA that create a gate for the transition of the another DNA strand, whereas type II topoisomerases cut the double strands of DNA and transit another duplex DNA through the gate and then both type I and type II topoisomerases religate the broken DNA strands [45]. Both the type I and type II topoisomerases are involved in the maintenance of the DNA topology. Inhibition of either type I or type II has significant impact on the DNA replication and other biological process. Expression level of topoisomerase II is often elevated in cancer cells [46]. Therefore, topoisomerase II is an attractive target for various antitumor and antimicrobial drugs [47]. The clinically successful/approved anticancer drugs which target the topoisomerase II induce topoisomerase II poisoning that leads to the arrest of replication and formation of duplex DNA break as the result cells undergo apoptosis [46]. Previous studies revealed that terpenes and terpenoids are potential inhibitor of topoisomerase II [47, 48] and they prefer to bind at the ATPase domain due to the hydrophobic nature of binding site [49]. Ursolic acid and thujone are lipophilic in nature. Thus, in the present study, topoisomerase II ATPase region was selected as a target.

**Figure 3:**
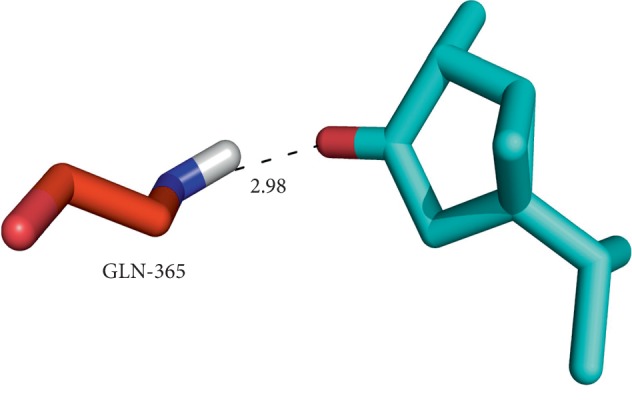
Binding of thujone at the interface of topoisomerase II (chain A) and the corresponding interactions with the residues.

Ursolic acid and thujone possess a significant binding affinity with topoisomerase II. The H-bond interactions between the ligands and proteins are N–H···O and O–H···O type. Besides the H-bond interactions, van der Waals and hydrophobic interactions with the ATPase region residues of topoisomerase II were noticed. The results of ursolic acid and thujone were compared with the docked pattern of CDX (cocrystallized ligand). The GLIDE energy of ligands, H-bond interactions along with distance, and docking score are given in Table 8. Ursolic acid showed H-bond interactions with the active site Thr27, Asn142, and Phe362 residues of a single chain of topoisomerase II (Figure 3) along with other nonbonded interactions with a sustainable docking score (−4.29) and GLIDE energy (−37.82 kcal/mol).

**Figure 4:**
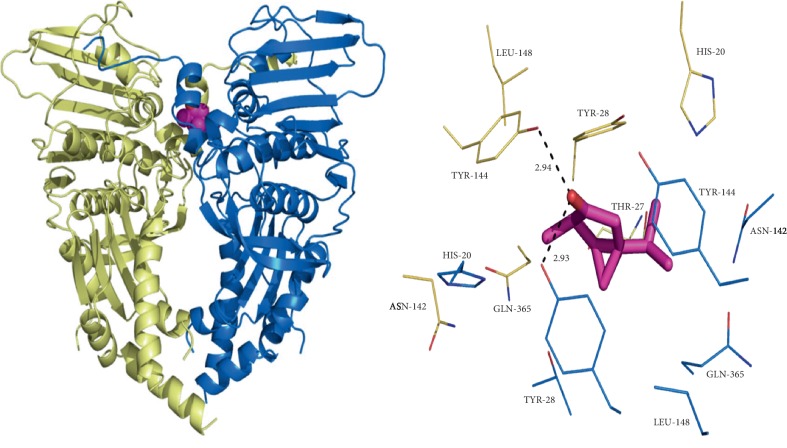
Binding of thujone at the interface of topoisomerase II (double chain) and the corresponding interactions with the residues.

**Figure 5:**
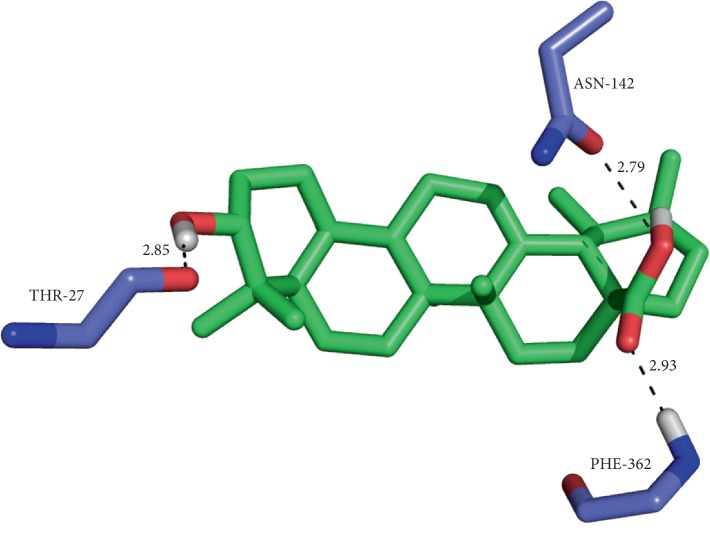
The interactions exhibited by ursolic acid with the active residues of topoisomerase II (chain A) and the corresponding interactions with the residues.

**Figure 6:**
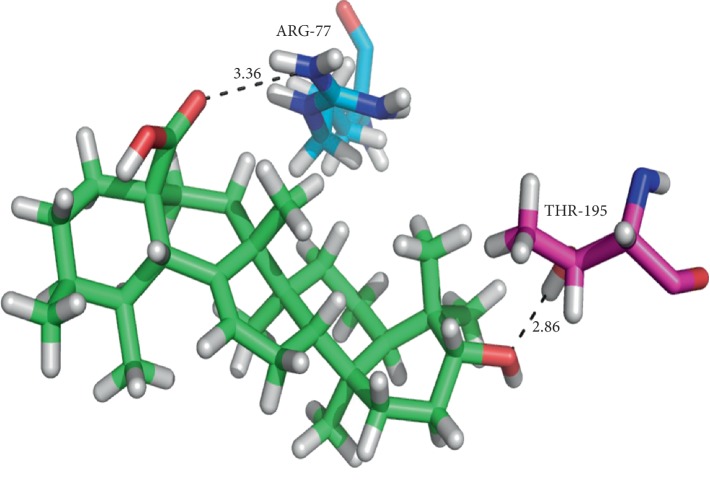
The binding of ursolic acid at the interface of topoisomerase II (double chain) and the corresponding interactions with the residues.

Moreover, while ursolic acid docked with both A and B chains displayed H-bond interactions with the Arg77a and Thr195b residues with notable GLIDE energy (−51.86 kcal/mol) and docking score (−8.0312) (Figure 4), thujone expressed H-bond interaction with the Gln365 residue (Figure 5) when docked with a single chain of topoisomerase II along with hydrophobic interactions with a docking score of −5.44 and GLIDE energy −16.96 kcal/mol. While docking, the thujone with two chains of topoisomerase II displayed interactions with Tyr144a and Tyr28b residues (Figure 6) with a considerable docking score (−8.18) and GLIDE energy (−27.38 kcal/mol). The docking results clearly show that ursolic acid is a strong inhibitor of 1QZR and energetically similar to the CDX. Hence, the antiproliferative activity of ursolic acid might be persistent via the findings of molecular docking studies with respect to the inhibition of topoisomerase II.

There are no detailed data available on the docking studies of ursolic acid and thujone with topoisomerase II. Even though earlier reports revealed that ursolic acid has significant inhibition potential on topoisomerase II in *in vitro* and *in vivo* assays [50], no detailed data is available on their *in silico* interactions. Two docking studies report the binding energy of ursolic acid (used as control) with topoisomerase II ATPase site (1QZR) but do not show the interaction between them. Those studies reported −21.2 kcal/mol [48] and −57.7 kJ/mol [51] (equivalent to −13.79 kcal/mol) as a binding energy of ursolic acid with topoisomerase II which is 2.44- and 3.76-fold higher than the present study, respectively; and the possible reason for the difference in binding energy is variations in the protein preparation and docking platform. This is the firsthand report of the *in silico* interactions of ursolic acid and thujone at the ATPase region of topoisomerase II. The antiproliferative activity of ursolic acid is supported by the docking results which may be achieved due to the inhibition of topoisomerase II.

## 4. Conclusion

Overall outcome of this investigation concludes that ursolic acid isolated from *M*. *edule* harbors significant antioxidant and antiproliferative potential with low IC_50_ values than the thujone isolated from *E*. *indica*. HL-60 cells are more sensitive to ursolic acid than the U-936 cells. The outcome of docking results shows that the ursolic acid has a strong affinity with the ATPase active site of topoisomerase II. Ursolic acid might be used as a good molecular template in the discovery of novel and highly potent antioxidant and antiproliferative agents.

## Figures and Tables

**Table 1:** DPPH radical scavenging activity of ursolic acid and thujone.

Concentration (*µ*g/mL)	DPPH radical scavenging activity (% of inhibition)^*∗*^			
	Ursolic acid	Thujone	Ascorbic acid	BHA
20	11.92 ± 0.53^a^	19.00 ± 0.33^a^	53.22 ± 0.27^a^	65.42 ± 0.44^a^
40	37.33 ± 0.57^b^	31.51 ± 0.64^b^	61.12 ± 0.66^b^	69.77 ± 0.58^b^
60	41.36 ± 0.31^c^	49.05 ± 0.62^c^	73.68 ± 0.38^c^	74.08 ± 0.38^c^
80	55.96 ± 0.54^d^	63.25 ± 0.82^d^	79.80 ± 0.27^d^	79.94 ± 0.17^d^
100	76.92 ± 0.53^e^	72.79 ± 0.82^e^	83.53 ± 0.41^e^	84.73 ± 0.38^e^

^*∗*^The values are mean of triplicates with (±) standard deviation (mean ± S.D; *n* = 3). Different superscript letters (a–e) in column within treatments indicates significant differences (at *p* < 0.05) when subject to Tukey's multiple comparison test.

**Table 2:** FRAP activity of ursolic acid and thujone.

Concentration (*µ*g/mL)	FRAP (OD values)^*∗*^			
	Ursolic acid	Thujone	Ascorbic acid	BHA
20	0.543 ± 0.006^a^	0.084 ± 0.016^a^	0.305 ± 0.007^a^	0.885 ± 0.007^a^
40	0.827 ± 0.006^b^	0.366 ± 0.009^b^	0.593 ± 0.006^b^	1.360 ± 0.006^b^
60	1.037 ± 0.006^c^	0.923 ± 0.023^c^	0.988 ± 0.010^c^	2.133 ± 0.011^c^
80	1.357 ± 0.006^d^	1.078 ± 0.015^d^	1.505 ± 0.040^d^	2.510 ± 0.009^d^
100	1.427 ± 0.170^d^	1.451 ± 0.005^e^	2.134 ± 0.012^e^	2.993 ± 0.005^e^

^*∗*^The values are mean of triplicates with (±) standard deviation (mean ± S.D; *n* = 3). Different superscript letters (a–e) in column within treatments indicate significant differences (at *p* < 0.05) when subject to Tukey's multiple comparison test.

**Table 3:** Hydroxyl radical scavenging activity of ursolic acid and thujone.

Concentration (*µ*g/mL)	Hydroxyl radical scavenging activity (% of inhibition)^*∗*^			
	Ursolic acid	Thujone	Ascorbic acid	BHA
20	46.59 ± 0.24^a^	26.43 ± 0.51^a^	49.60 ± 0.55^a^	42.75 ± 0.52^a^
40	53.48 ± 0.07^b^	47.02 ± 0.71^b^	61.40 ± 0.41^b^	49.65 ± 0.38^b^
60	60.20 ± 0.33^c^	53.35 ± 0.47^c^	76.43 ± 0.34^c^	60.18 ± 0.75^c^
80	70.51 ± 0.21^d^	56.76 ± 0.30^d^	88.25 ± 0.42^d^	73.15 ± 0.38^d^
100	74.62 ± 0.17^e^	63.34 ± 0.22^e^	94.06 ± 0.60^e^	88.55 ± 0.57^e^

^*∗*^The values are mean of triplicates with (±) standard deviation (mean ± S.D; *n* = 3). Different superscript letters (a–e) in column within treatments indicates significant differences (at *p* < 0.05) when subject to Tukey's multiple comparison test.

**Table 4:** Nitric oxide radical scavenging activity of ursolic acid and thujone.

Concentration (*µ*g/mL)	Nitric oxide radical scavenging activity (% of inhibition)^*∗*^			
	Ursolic acid	Thujone	Ascorbic acid	BHA
20	|21.70 ± 0.25^a^	14.25 ± 0.88^a^	28.80 ± 1.21^a^	30.26 ± 0.66^a^
40	32.86 ± 0.32^b^	29.61 ± 0.66^b^	54.53 ± 0.91^b^	41.23 ± 1.54^b^
60	40.16 ± 0.34^c^	39.55 ± 2.16^c^	65.79 ± 0.96^c^	53.58 ± 0.67^c^
80	59.49 ± 0.18^d^	51.24 ± 0.51^d^	79.39 ± 1.22^d^	65.35 ± 1.54^d^
100	70.50 ± 0.39^e^	74.12 ± 0.66^e^	94.15 ± 1.13^e^	87.28 ± 0.38^e^

^*∗*^The values are mean of triplicates with (±) standard deviation (mean ± S.D; *n* = 3). Different superscript letters (a–e) in column within treatments indicates significant differences (at *p* < 0.05) when subject to Tukey's multiple comparison test.

**Table 5:** Superoxide radical scavenging activity of ursolic acid and thujone.

Concentration (*µ*g/mL)	Superoxide radical scavenging activity (% of inhibition)^*∗*^			
	Ursolic acid	Thujone	Ascorbic acid	BHA
20	24.11 ± 1.13^a^	07.95 ± 0.71^a^	25.31 ± 0.29^a^	13.04 ± 0.62^a^
40	48.49 ± 0.41^b^	10.95 ± 0.37^a,b^	34.24 ± 0.42^b^	24.10 ± 0.45^b^
60	57.71 ± 0.89^c^	19.82 ± 0.58^a,b^	44.62 ± 0.54^c^	37.28 ± 0.17^c^
80	64.67 ± 0.41^d^	24.64 ± 0.37^b^	60.06 ± 0.50^d^	40.18 ± 0.42 ^d^
100	76.91 ± 0.33^e^	43.41 ± 9.69^c^	75.56 ± 0.25^e^	56.35 ± 0.29^e^

^*∗*^The values are mean of triplicates with standard deviation (mean ± S.D; *n* = 3). Different superscript letters (a–e) in column within treatments indicates significant differences (at *p* < 0.05) when subject to Tukey's multiple comparison test.

**Table 6:** Antioxidant activities IC_50_/EC_50_ values of ursolic acid and thujone.

Assays	IC_50_/EC_50_ values (*μ*g/mL)^#^			
	Ursolic acid	Thujone	Ascorbic acid	BHA
DPPH radical	71.86 ± 0.54	60.92 ± 1.61	18.77 ± 0.52	15.48 ± 0.61
FRAP	18.42 ± 0.03	44.58 ± 0.89	33.62 ± 0.61	11.24 ± 0.37
Hydroxyl radical	29.69 ± 0.44	49.47 ± 0.62	20.47 ± 0.86	40.78 ± 0.98
Nitric oxide radical	70.40 ± 0.08	77.68 ± 0.58	36.43 ± 0.71	53.90 ± 0.64
Superoxide radical	43.35 ± 0.95	131.78 ± 1.27	67.03 ± 0.80	90.84 ± 1.76

^#^The values are mean of triplicates with standard deviation (mean ± S.D; *n* = 3).

**Table 7:** Antiproliferative activity of ursolic acid and thujone on U-937 and HL-60 cell lines.

Concentration (*µ*mol/mL)	% Cell viability^*∗*^			
	Ursolic acid		Thujone	
	U-937 CL^#^	HL-60 CL^#^	U-937 CL^#^	HL-60 CL^#^
03.13	73.15 ± 2.01^h^	69.64 ± 0.68^h^	90.17 ± 1.08^h^	96.33 ± 0.78^h^
06.25	65.18 ± 1.86^g^	66.92 ± 1.31^h^	85.80 ± 0.64^g^	88.88 ± 0.91^g^
12.50	59.63 ± 0.67^f^	56.29 ± 0.75^g^	78.38 ± 1.64^f^	83.22 ± 1.10^f^
25.00	54.69 ± 0.64^e^	50.44 ± 0.79^f^	71.38 ± 1.03^e^	77.48 ± 1.66^e^
50.00	44.66 ± 1.35^d^	45.18 ± 0.75^e^	69.67 ± 0.68^e^	71.15 ± 1.23^d^
100.0	32.74 ± 2.58^c^	39.01 ± 1.17^d^	64.59 ± 1.20^d^	56.22 ± 0.70^c^
250.0	27.67 ± 1.28^b^	33.37 ± 1.17^c^	53.85 ± 1.80^c^	52.66 ± 1.52^b^
500.0	23.08 ± 1.65^a^	29.15 ± 0.55^b^	42.51 ± 0.73^b^	49.67 ± 0.69^b^
1000	19.67 ± 0.98^a^	23.70 ± 1.27^a^	37.29 ± 1.43^a^	44.66 ± 1.26^a^

Control, nil mortality. ^*∗*^The values are mean of triplicates with standard deviation (mean ± S.D; *n* = 3). Different superscript letters (a–i) in column within treatments indicates significant differences (at *p* < 0.05) when subject to Tukey's multiple comparison test, CL^#^, cell line.

**Table 8:** Docking results of ursolic acid and thujone with topoisomerase II (1QZR).

Compounds	Docking score	GLIDE energy (kcal/mol)	Interactions D–H···A	D···A^*∗*^ distance (Å)
*Target protein: 1QZR (single chain)*				
Ursolic acid	−4.2933	−37.82	(O–H···O) ASN142	2.79
			PHE362 (N–H···O)	2.93
			(O–H···O) THR27	2.85
Thujone	−5.4444	−16.964	GLN365 (N–H···O)	2.98
			TYR28 (O–H···O)	3.05
			GLN365 (N–H···N)	3.15
CDX (cocrystallized ligand)	−7.0166	−35.33	(O–H···O) THR363	3.30
			(N–H···O) THR363	3.16
			(N–H···O) PHE362	3.32
			(O–H···O) PHE362	2.80
			PHE362 (N–H···O)	3.46
*Target protein: 1QZR (double chain)*				
Ursolic acid	−8.0312	−51.86	(N–H···O) ARG77A	3.36
			THR195B (O–H···O)	2.86
Thujone	−8.1810	−27.38	TYR144A (O–H···O)	2.94
			TYR28B (O–H···O)	2.93
			(O–H···O) THR27A	
CDX (cocrystallized ligand)	−12.105	−56.86	GLN365A (O–H···N)	3.24
			GLN365A (O–H···N)	2.74
				2.73

^*∗*^D, donor; A, acceptor.

## Data Availability

No data were used to support this study.
